# Beyond the “Pain Matrix,” inter-run synchronization during mechanical nociceptive stimulation

**DOI:** 10.3389/fnhum.2014.00265

**Published:** 2014-05-05

**Authors:** Franco Cauda, Tommaso Costa, Matteo Diano, Sergio Duca, Diana M. E. Torta

**Affiliations:** ^1^GCS fMRI, Koelliker Hospital and Department of Psychology, University of TurinTurin, Italy; ^2^Department of Psychology, University of TurinTurin, Italy

**Keywords:** fMRI, pain measurement, inter-run synchronization, imaging, GLM

## Abstract

Pain is a complex experience that is thought to emerge from the activity of multiple brain areas, some of which are inconsistently detected using traditional fMRI analysis. One hypothesis is that the traditional analysis of pain-related cerebral responses, by relying on the correlation of a predictor and the canonical hemodynamic response function (HRF)- the general linear model (GLM)- may under-detect the activity of those areas involved in stimulus processing that do not present a canonical HRF. In this study, we employed an innovative data-driven processing approach- an inter-run synchronization (IRS) analysis- that has the advantage of not establishing any pre-determined predictor definition. With this method we were able to evidence the involvement of several brain regions that are not usually found when using predictor-based analysis. These areas are synchronized during the administration of mechanical punctate stimuli and are characterized by a BOLD response different from the canonical HRF. This finding opens to new approaches in the study of pain imaging.

## Introduction

Pain is a multidimensional phenomenon that is thought to emerge from the activity of a number of sensory, cognitive, and emotional cortical circuits (Apkarian et al., 2005; Friebel et al., 2011). The cortical and subcortical areas related to pain processing have been extensively studied using functional magnetic resonance (fMRI) and positron emission tomography (PET). However, as evidenced by three meta-analyses published in the last 10 years, there is no agreement among studies regarding brain activations in response to painful stimuli. Peyron et al. (2000) concluded that the majority of the studies reported activations to painful stimuli in the secondary somatosensory cortices, the anterior cingulate cortex and the insular cortex and less consistently in the thalamus and primary somatosensory cortex. Apkarian et al. (2005) showed that six areas commonly respond to painful stimuli: the secondary somatosensory cortex, the anterior cingulate cortex, the insular cortex, but also the thalamus, the primary somatosensory cortex and prefrontal regions. Finally, Friebel et al. (2011), by conducting a coordinate-based meta-analysis, showed that painful stimuli activate the bilateral secondary somatosensory cortex, the mid-cingulate cortex, the right inferior parietal lobe, the bilateral thalamus, the anterior insula and the supplementary motor areas. Other regions, such as the primary motor cortex and the temporal cortex, the cerebellum, the amygdala, the parabrachial nucleus, and the periaqueductal gray have been less frequently observed (Peyron et al., 2000; Derbyshire, 2003; Strigo et al., 2003; Apkarian et al., 2005) during fMRI recordings. The functional meaning of these responses to painful stimuli is debated and recent views suggest that they may reflect the activity of saliency detectors or multimodal processing (Downar et al., 2002; Cauda et al., 2011; Legrain et al., 2011; Mouraux et al., 2011; Lotsch et al., 2012). Inconsistent activations of brain areas in response to painful stimuli have been mainly attributed to characteristics of the task, cognitive factors, stimulus type and stimulus location (see Apkarian et al., 2005, for a discussion on this point).

However, an interesting but unexplored possibility is that the variability in the identification of which areas respond to nociceptive and painful stimuli may be explained also by methodological factors. With classical approaches, painful or nociceptive stimuli are presented to volunteers during fMRI acquisition and a set of predictors is created. These predictors are then subsequently convolved with a response function and voxelwise correlated with the measured time courses (Friston et al., 1995). This kind of analysis relies on the assumption of a spatially and temporally replicable BOLD function. However, it has been shown that this approach leads to an underestimation of the number of areas that are involved in the processing of the incoming stimulus. This may be due to two different problems: (i) the signal may have a low signal-to-noise ratio and (ii) all areas characterized by a hemodynamic function other than the HRF are not well-modeled with a gamma-like predictor. In a recent paper, (Gonzalez-Castillo et al., 2012) it has been shown that, under optimal conditions, nearly the whole cortex (96%) responds to external stimuli with stimulus-locked significant BOLD variation. Crucially, almost 68% of the reported 96% activations are due to responses other than the canonic hemodynamic function.

In this study we have investigated whether methods that do not impose an a priori gamma-like function may identify activations in areas that are not characterized by a canonical HRF. These activations may be not observed when using classical methodological approaches. Participants received mechanical punctate stimuli during an fMRI session; cerebral activations were measured by using an inter-run synchronization (IRS) approach. The idea behind this method was first proposed by Hasson et al. (2004). It assumes that synchronous activity of a brain region in all replications of the same stimulation (e.g., in all trials in the same subject) reflects the involvement of that area in the processing of the stimuli. This method has demonstrated good reliability (Seghier and Price, 2009; Hasson et al., 2010) for detecting time-locked activity in brain areas, including some areas that are not reliably identified in most conventional fMRI protocols. Importantly, this method does not guarantee the complete detection of all the stimulus-locked brain areas but allows increased sensitivity in the detection of activity in areas that respond with non-conventional BOLD envelopes. The IRS approach does not include any a priori hypothesis and is thus particularly suited to discover the involvement of those brain areas whose activation may be underestimated by traditional fMRI approaches.

## Materials and methods

### Participants

Seventeen healthy right-handed volunteers (8 women mean age 28 ± 4.2) participated in the study. Participants were free of neurological or psychiatric disorders, not taking any psychoactive medication, and did not present a history of drug or alcohol abuse. We obtained the written informed consent of each subject, in accordance with the Declaration of Helsinki. The study was approved by the institutional committee on ethical use of human subjects at the University of Turin.

### Task and image acquisition

We performed a slow event-related paradigm composed of four different runs. In each run, participants received 24 stimuli either on their right or left hand (12 stimuli on the left and 12 stimuli on the right). Mechanical punctate stimuli were applied with a hand-held 256 mN pinprick probe stimulator, which preferably activates high-threshold mechanoreceptors and type-I mechano-heat receptors (Magerl et al., 2001).

In each run, the inter-stimulus interval (ISI) ranged pseudo randomly between 18 and 22 s. The stimulation site was changed slightly after each stimulus and no more than three consecutive stimuli were applied to the same hand. Each run was different from the other, meaning that the order of presentation of the stimuli on the left and right hands and the ISI between stimuli changed in every run.

Images were obtained using a 1.5 Tesla INTERA™ scanner (Philips Medical Systems). We acquired three-dimensional high-resolution T_1_-weighted structural images using a Fast Field Echo (FFE) sequence, with a 25 ms TR, an ultra-short TE, and a 30° flip angle. The acquisition matrix was 256 × 256, and the FoV was 256 mm. The set consisted of 160 contiguous sagittal images covering the whole brain.

Functional T2^*^ weighted images were acquired using echoplanar (EPI) sequences, with a repetition time (TR) of 2000 ms, an echo time (TE) of 50 ms, and a 90° flip angle. The acquisition matrix was 64 × 64, with a 200 mm field of view (FoV). A total of 240 volumes were acquired, with each volume consisting of 19 axial slices; slice thickness was 4.5 mm with a 0.5 mm gap. The first two scans were discarded directly from the scanner.

In order to investigate if brain activations were specific for the mechanical stimulus, we performed a control experiment using tactile stimuli applied using a SenseLab™Brush-05. A second cohort of 12 healthy right-handed volunteers (6 females mean age 29 ± 7.41 years) took part in the control experiment in which we adopted exactly the same design as before.

### Data analysis

The data-sets were pre-processed and analyzed using the BrainVoyager QX software (Brain Innovation, Maastricht, The Netherlands). We corrected the intensity of the acquired measures using a mean intensity adjustment. For each volume, we computed the average intensity across the first image, for each subsequent scan of the same volume, we computed the mean intensity and then scaled to obtain the same average volume intensity. A 3D motion correction was used to correct for small head movements. All volumes were aligned spatially to the first volume by rigid body transformations, using a trilinear-sinc interpolation algorithm. We employed a slice scan time correction to allow a whole volume to be treated as a single data point. The sequentially scanned slices comprising each volume were interpolated in time, using a signal sinc-interpolation algorithm. Spatial data smoothing was performed using a 3D Gaussian kernel with full-width half maximum of 8 mm. We also applied linear and non-linear trend removal using a high pass filter (*f* < 3 cycles in time course). For all temporal analyses no temporal smoothing or low-pass filter was applied to preserve the temporal characteristics of the signal. Temporal smoothing of 2.8 s FWHM was only applied for the GLM analysis.

Each subject's slice-based functional scan was co-registered with their 3D high-resolution structural scan. Subsequently, we transformed the 3D structural data-set of each subject into Talairach space the cerebrum was translated and rotated into the anterior-posterior commissure plane and the borders of the cerebrum were identified.

We used an anatomical-functional coregistration to transform into Talairach space the functional time course of each subject and to create the volume time course.

### Inter-run synchronization (IRS)

To compute the IRS we modified the methodology first introduced by Hasson et al. (2004). Our method included two steps of analysis, one at the single subjective level (step 1) and one at the group level (step 2). At step 1, (single subject level), we used the z-normalized time courses of each voxel in the four runs to derive a series of similarity measures. That is, we obtained a value of how much the activity of a voxel in one run correlated with its activity in each of the other three runs. Pearson's correlation coefficient was used to calculate correlation values. Indeed for each run we calculated the voxelwise temporal correlation between homologous voxels. More specifically, for a given voxel, we calculated the correlation of its normalized time course across all the possible permutations of the four runs. For each subject, we correlated the activations of the first run with those of the second, third and fourth run and so forth for all the six permutations. For each correlation a statistical map was created. In this way, we obtained six correlation maps for each subject. Significance levels were corrected for multiple comparisons using the False Discovery Rate (FDR). These maps were then r-to-z transformed using Fisher's r-to-z transformation.

To summarize: at the subject level we performed a one-sample *t*-test on the six r-to-z transformed maps and we obtained a single cumulative subject map. Significance levels were corrected for multiple comparisons using the FDR. In the second step (multi-subject level), we summarized all the subject-specific maps using a one-sample *t*-test. Significance levels were corrected for multiple comparisons using the FDR. As a comparison, we also calculated the conventional multi-subject statistical analysis using the general linear model (GLM) and a random effect procedure.

In order to perform the GLM analysis, a single design matrix was specified for all subjects in each task condition, consisting of task-defined box-car time courses convolved with a pre-defined hemodynamic response function (HRF) (Boynton et al., 1996) to account for the hemodynamic delay. This design matrix was then entered into subject-level GLM analyses to yield beta parameter estimates for subsequent group statistics. At the group level, a one sample *t*-test was performed between all subject-specific maps. All the analyses were thresholded with a *q* < 0.05, FDR corrected. Event-related averages were calculated to show the temporal profile of activation in specific regions. In addition, a conjunction test was performed to investigate voxels that were simultaneously activated by the IRS- and GLM-based analysis. To do so, we attributed a value of 1 to each active voxel of each multi subject map (one for GLM and one for IRS; threshold of *q* < 0.05, FDR corrected). We then summed the two maps and reported in Figure 3 only voxels with a value >1, therefore voxels that were activated by both methods.

A winner-take all map was calculated to detect the region in which the signal generated by one of our two techniques was maximal. We attributed to each brain voxel a value (0 for IRS and 1 for GLM) depending on which method generated a greater statistical value in that voxel (multi-subjects summary statistics). We colored voxels with a 0 value (IRS) in green and voxels with a value of 1 (GLM) in red.

## Results

**Figure 1:**
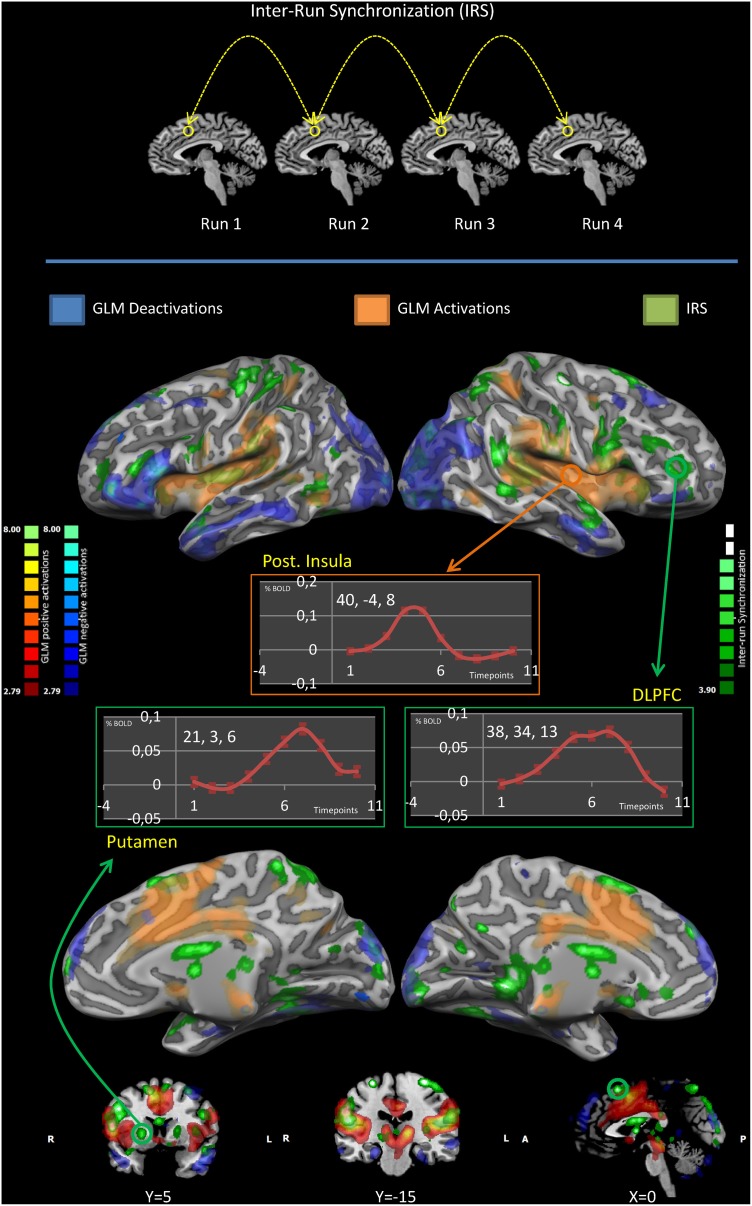
**IRS and GLM results**. The upper panel illustrates the idea of the inter-run synchronization (IRS) approach: areas that are synchronous in all replications of the same stimulation (in the same subject) are likely to be involved in the processing of the stimuli and will be the only commonality among runs. The middle and lower panels summarize the IRS and GLM results. Results are significant at *p* < 0.05. Significance levels were corrected for multiple comparisons using the false discovery rate (FDR). GLM deactivations are colored in blue, GLM activations in red, IRS activations in green. Event-related averages of the BOLD response in the posterior insula, putamen and the dorsolateral prefrontal cortex are shown. Please note only the posterior insula has a canonic HRF.

The results of the IRS overlapped with GLM results in several areas, both for activations and deactivations (see Figure 1). In addition, broader activations encompassing the prefrontal, premotor, sensorimotor, posterior parietal, basal ganglia, hypothalamus and cerebellar areas were identified when using the IRS approach. (Figure 1 and Table 1).

**Table 1:** **Significant IRS areas**.

**Area**	**Voxels**	**L/R%**	**Left BA**	**Right BA**
Bilateral precuneus	31188	57/43%	BA 7, BA 19, BA 6, BA 10	BA 7, BA 19, BA 6, BA 10
Bilateral posterior cingulate	597	66/34%	BA 29, BA 23, BA 30	BA 29, BA 23, BA 30, BA 31
Bilateral anterior cingulate	920	53/47%	BA 32, BA 24, BA 10, BA 33	BA 32, BA 24, BA 10, BA 33
Bilateral transverse temporal gyrus	179	81/19%	BA 41	BA 41
Bilateral fusiform gyrus	3105	48/52%	BA 37, BA 19, BA 18, BA 20	BA 37, BA 19, BA 18, BA 20
Bilateral inferior occipital gyrus	1022	17/83%	BA 18, BA 17	BA 18, BA 17, BA 19
Bilateral inferior temporal gyrus	532	99/1%	BA 37, BA 19, BA 21	BA 37, BA 20
Bilateral insula	3875	86/14%	BA 13, BA 40, BA 41, BA 44	BA 13, BA 40, BA 41, BA 42
Bilateral parahippocampal gyrus	3005	56/44%	BA 30, BA 19, BA 37, BA 27	BA 30, BA 19, BA 37
Bilateral lingual gyrus	4483	60/40%	BA 17, BA 18, BA 19, BA 30	BA 17, BA 18, BA 19, BA 30
Bilateral middle occipital gyrus	2373	27/73%	BA 18, BA 19, BA 37	BA 18, BA 19, BA 39
Bilateral middle temporal gyrus	5924	51/49%	BA 21, BA 22, BA 37, BA 39	BA 21, BA 22, BA 39
Bilateral superior temporal gyrus	8560	40/60%	BA 22, BA 41, BA 42, BA 13	BA 22, BA 41, BA 42, BA 13
Bilateral inferior frontal gyrus	3733	37/63%	BA 9, BA 44, BA 45, BA 47	BA 9, BA 44, BA 45, BA 6
Bilateral cuneus	5653	28/72%	BA 19, BA 18, BA 17	BA 19, BA 18, BA 17, BA 31
Right supramarginal gyrus	895	0/100%	–	BA 40
Bilateral cingulate gyrus	595	75/25%	BA 23, BA 31, BA 32, BA 24	BA 23, BA 31, BA 32
Bilateral inferior parietal lobule	5206	62/38%	BA 40, BA 13, BA 2	BA 40, BA 7, BA 13, BA 39
Bilateral precuneus	5353	12/88%	BA 7, BA 19, BA 31	BA 7, BA 19, BA 31
Bilateral superior parietal lobule	3059	11/89%	BA 7, BA 5	BA 7, BA 5, BA 40
Bilateral middle frontal gyrus	3171	70/30%	BA 6, BA 10, BA 8	BA 6, BA 9, BA 8
Bilateral paracentral lobule	273	34/66%	BA 4, BA 6	BA 5, BA 4, BA 3, BA 7
Bilateral post-central gyrus	9941	58/42%	BA 2, BA 3, BA 40, BA 5	BA 2, BA 3, BA 40, BA 5
Bilateral pre-central gyrus	5925	56/44%	BA 6, BA 4, BA 44, BA 9	BA 6, BA 4, BA 44, BA 9
Bilateral superior frontal gyrus	7474	70/30%	BA 6, BA 8, BA 10, BA 9	BA 6, BA 8, BA 10
Bilateral medial frontal gyrus	2892	55/45%	BA 10, BA 6, BA 9, BA 8	BA 10, BA 6, BA 8
Bilateral tuber of vermis	141	46/54%	–	–
Bilateral declive of vermis	142	49/51%	–	–
Bilateral cerebellar tonsil	2026	42/58%	–	–
Bilateral inferior semilunar lobule	1872	30/70%	–	–
Bilateral nodule	138	54/46%	–	–
Bilateral uvula	150	6/94%	–	
Bilateral pyramis	1120	9/91%	–	–
Bilateral tuber	469	10/90%	–	–
Bilateral declive	3205	41/59%	BA 19, BA 18	BA 19, BA 37
Bilateral culmen	4483	50/50%	BA 19, BA 30, BA 37	BA 19, BA 37
Bilateral hippocampus	393	43/57%	–	–
Bilateral hypothalamus	192	8/92%	–	–
Right substantia nigra	154	0/100%	–	–
Bilateral caudate body	1229	60/40%	–	–
Bilateral ventral anterior nucleus	329	60/40%	–	–
Bilateral ventral posterior lateral nucleus	322	57/43%	–	–
Bilateral medial dorsal Nucleus	308	19/81%	–	–
Bilateral pulvinar	1212	50/50%	–	–
Bilateral ventral lateral nucleus	659	19/81%	–	–
Bilateral anterior nucleus	430	28/72%	–	–
Right mammillary body	129	0/100%	–	–
Bilateral medial globus pallidus	514	18/82%	–	–
Bilateral lateral globus pallidus	733	48/52%	–	–
Bilateral putamen	3691	92/8%	–	–

*Areas significantly correlated between painful stimulation runs. P < 0.05, Significance levels corrected for multiple comparisons using the False Discovery Rate (FDR)*.

In the putamen and in the dorsolateral prefrontal cortex the BOLD signal was characterized by a time course markedly different from the canonical HRF (Glover, 1999). Conversely, the time course of the BOLD activity in the posterior insula corresponded to the canonical HRF.

**Figure 2:**
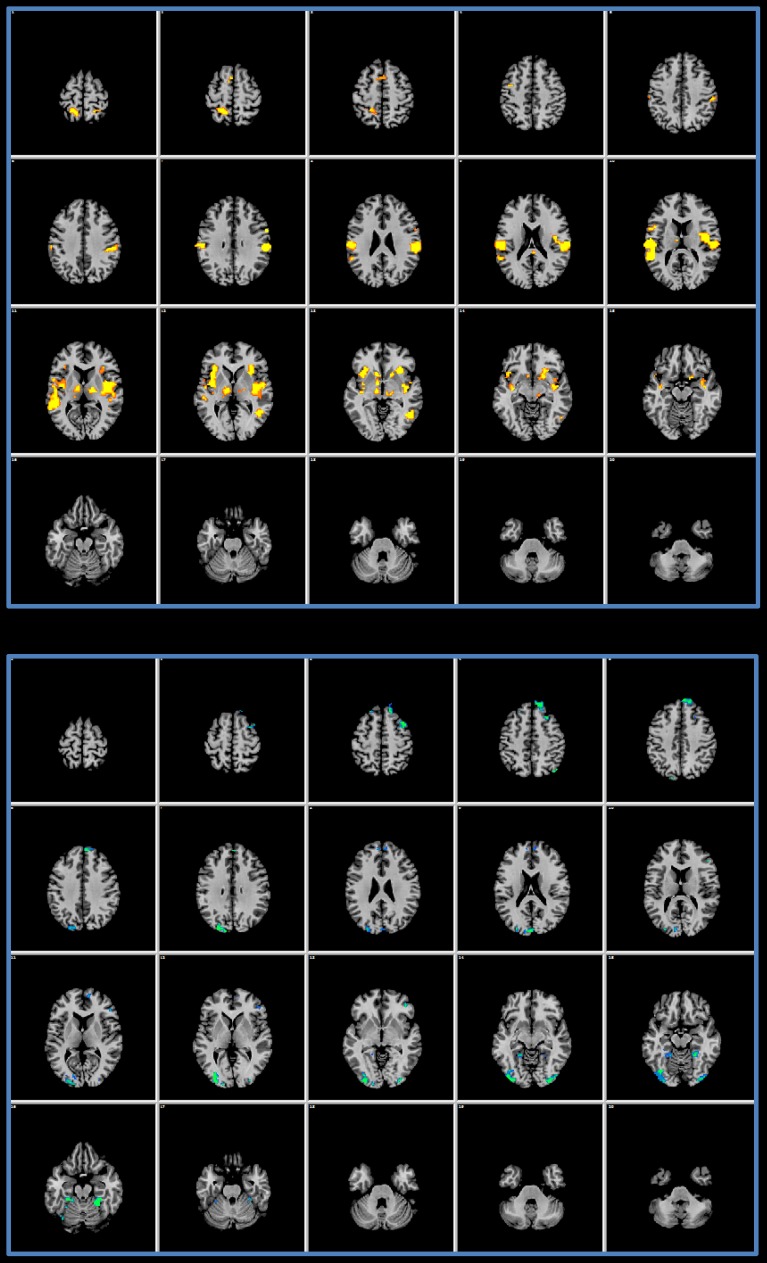
**Conjunction analysis**. The upper panel shows the results of a conjunction analysis of GLM activations and IRS results. In the lower panel the conjunction of the GLM deactivations and of the IRS is presented.

Figure 2 offers the details of the convergence between the IRS and the GLM results: we performed a conjunction analysis between the IRS results and the GLM activations (upper panel) and deactivations (lower panel). It can be noted that IRS and GLM deactivations overlap to a smaller extent as compared to activations.

**Figure 3:**
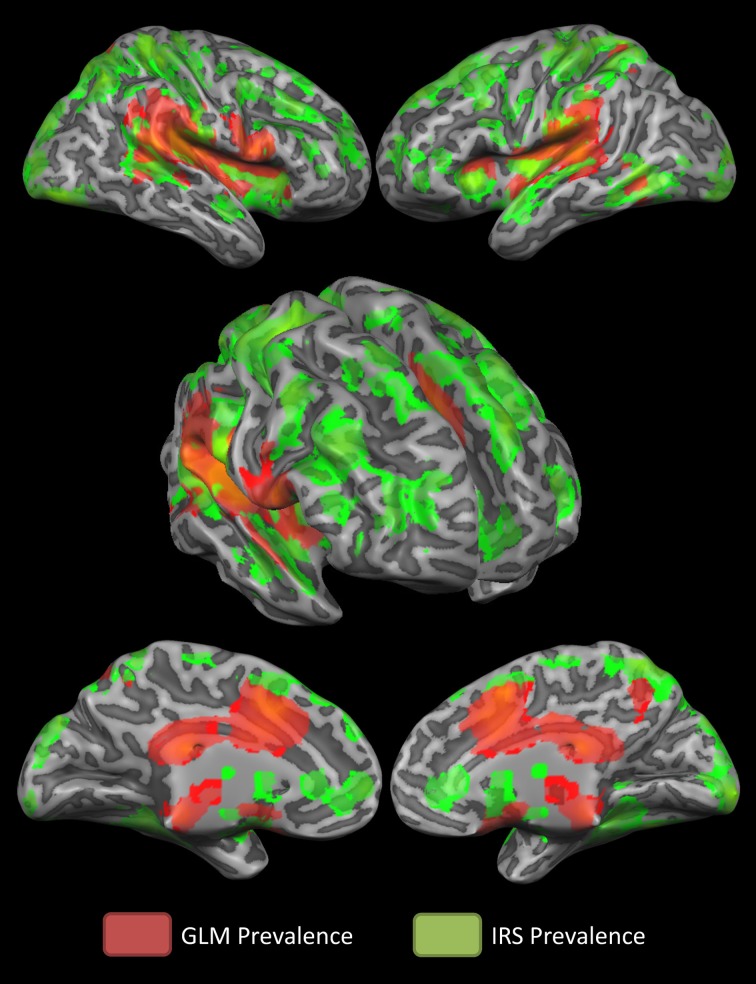
**Winner-take- all map**. The winner- take- all map shows, for active regions, which technique detected activations better.

Figure 3 summarizes which technique is more suitable to detect activity in specific brain regions. It is shown that the GLM captures activity in the mid and posterior cingulate cortex better than the IRS which, conversely, detects better activations of the anterior insula and the anterior cingulate cortex.

**Figure 4:**
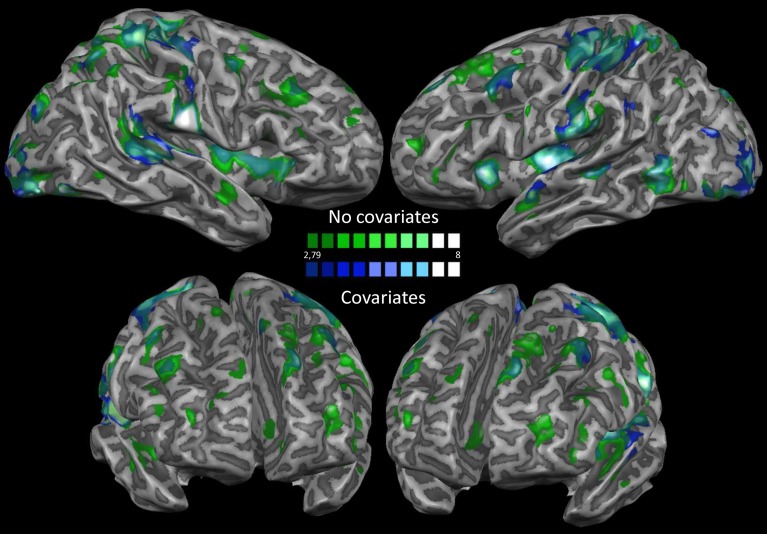
**Nuisance regression**. This map shows a comparison between the results obtained before and after removing motion, white matter and cerebrospinal fluid covariates from the dataset.

In order to exclude the effect of physiological artifacts on our data, we also performed the same analyses using motion, white matter (WM) and cerebrospinal fluid (CSF) as covariates. We show the difference between the two analyses in Figure 4. As it can be seen, differences were minimal, therefore confirming that the nuisances were not biasing significantly our results.

**Figure 5:**
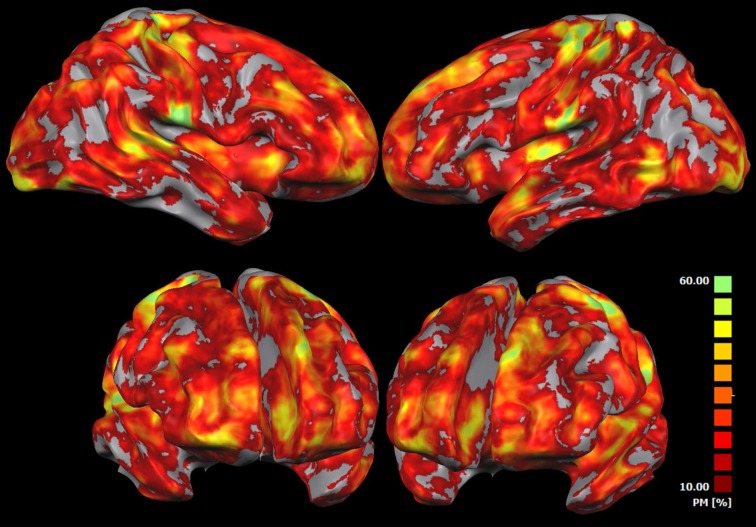
**IRS results variability**. This map shows the probability that each voxel has to be found active in one or more subjects when using the IRS. At each spatial location, such maps represent the relative number of subjects reporting a significant IRS activation. The probability map is calculated by summing voxel value of each subject-related IRS result and dividing this value by the number of subjects.

Figure 5 shows the probability that a voxel is activated in one or in more subjects (IRS analysis). At each spatial location, the map represents the number of subjects showing significant activations. This map indicates that significant areas of activations obtained with the IRS can be observed in >60% of the participants.

**Figure 6:**
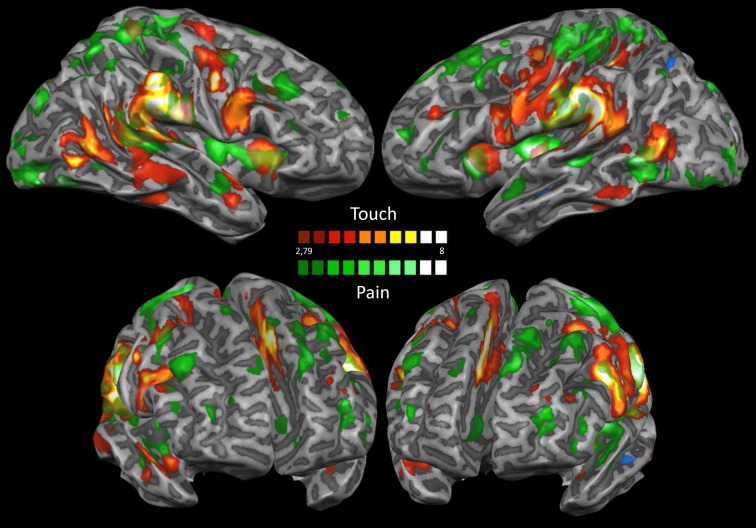
**IRS mechanical punctate and tactile stimulations**. This map shows an overlay of the IRS following tactile and nociceptive stimulation. Results are significant at *p* < 0.05; significance levels were corrected for multiple comparisons using the False discovery rate (FDR). Activations elicited by nociceptive stimulation are shown in green, activations elicited by tactile stimulation are colored in red.

Figure 6 shows an overlay of the activations in response to mechanical punctate and tactile stimuli (IRS). Several areas of overlap can be observed. However, it can also be seen that the posterior insula and the anterior cingulate cortex were only activated by mechanical punctate stimulation and not by tactile stimuli.

## Discussion

In this paper we show, using an innovative technique, that classical techniques, such as the GLM, which convolve a predictor with the canonic HRF, may lead to the underestimation of cerebral responses to mechanical punctate stimuli. Therefore, we propose that previous inconsistencies in the reported cerebral activations to painful stimuli might have been due to methodological approaches and not only to differences in cognitive factors, stimulus kind and experimental design (Apkarian et al., 2005). Using a non-conventional approach, the IRS, we were able to show that several areas, inconsistently identified when using a classical predictor-based analysis, activate in response to a mechanical punctate stimulus. This result suggests that the set of areas that participate in the processing of painful stimuli may be more extensive than what is usually found using predictor-based analysis with fixed hemodynamic responses. Our results suggest that several areas that activate in response to painful stimuli are characterized by a non-canonical BOLD response (Glover, 1999) and are, for this reason, incompletely or inconsistently detected using analysis that assume fixed underlying hemodynamic responses.

We observed that the GLM and the IRS identified different areas of activation in response to the external stimuli. Areas with a canonical shape of the BOLD response were better identified with the GLM, whereas areas showing a non-canonical HRF were only observed when the IRS was used. By performing a control analysis using physiological noise as covariate, we also excluded that the activations observed when using the IRS were mainly artifact-driven. This spatial variability of the BOLD response (Saad et al., 2001; Chang et al., 2008) can be related to the different temporal profile of intervention of different areas (Pichè et al., 2010) or brain networks in the elaboration of noxious inputs (Cauda et al., 2013a; Mayhew et al., 2013). Indeed painful stimulation elicits a series of responses in a widespread pattern of brain areas, whose functional significance is not completely understood yet (Mouraux and Iannetti, 2009; Legrain et al., 2011; Mouraux et al., 2011; Cauda et al., 2012).

We compared the sensitivity of each method in detecting brain activations in response to the stimuli. Our results showed that the IRS detected activations in the prefrontal and premotor regions, the posterior parietal cortex, the basal ganglia and the cerebellum in response to mechanical punctate stimuli. These areas are not frequently reported in fMRI studies of pain. Besides, the IRS was more able to identify activity in the anterior cingulate cortex. Therefore, it may be concluded that the activity in the aforementioned areas was significantly correlated across runs. The IRS however, failed to identify any activation in the anterior insula and mid-cingulate cortex, two areas that are commonly observed in pain-related tasks and that were detected by the GLM analysis. How to explain that the IRS failed to identify activations in areas usually detected in pain research? One possibility is that although studies with intracranial recordings have proposed that neurons in the mid-cingulate cortex respond specifically to nociceptive stimuli (Frot et al., 2008), there is also convincing evidence that the anterior insula and the mid-cingulate cortex are not specifically activated by nociceptive and painful such stimuli (Legrain et al., 2011; Mouraux et al., 2011; Shackman et al., 2011; Torta and Cauda, 2011; Yarkoni et al., 2011). Indeed, these two areas participate in multisensory and multimodal processing and in saliency detection (Downar et al., 2002; Cauda et al., 2011, 2012; Legrain et al., 2011; Mouraux et al., 2011; Torta and Cauda, 2011; Lotsch et al., 2012; Torta et al., 2013) and their activation is related to an evaluative process that involves the integration of several different homeostatic, proprioceptive, hedonic and environmental information. Furthermore these areas are involved in autonomic regulation (Critchley et al., 2000; Craig, 2002, 2009, 2010; Critchley, 2005; Dube et al., 2009; Cauda et al., 2012, 2013b) and their activity has been shown to drive trial-to-trial variability in the anterior insula (Pichè et al., 2010). Some meta-analytic data (Cauda et al., 2012; Torta et al., 2013) support the possibility that the anterior insula and mid-cingulate cortex constitute hub areas devoted to the exchange of information among large–scale brain network. Thus, it may be suggested that the failure of the IRS to detect activations in these multimodal areas may have been driven by increased variability, which is not observed in areas univocally modulated by the task. Indeed, some studies have proposed that the anterior insula and the executive network are characterized by a high degree of internal memory and maintain a stronger background of auto-correlated activity (Kaneoke et al., 2012). Conversely, input areas like S1 are more prone to be modulated efficiently by external stimuli. The auto-correlated activity in the insula and in the executive network is less likely to correlate between runs and therefore leads to less reliable results of the IRS. Indeed, the IRS captures best those activities that are correlated across runs. The activity in the insula and in the mid-cingulate cortex were also found to have a low level of inter-subject synchronization in other studies (Hasson et al., 2010).

By performing event-related averages of the BOLD signal in the posterior insula, the dorsolateral prefrontal cortex and the putamen, we observed that these three areas are characterized by a different shape of the response. The posterior insula, which is well-detected by traditional analysis, showed, as expected, an almost perfect gamma-like BOLD response. Conversely, the dorsolateral prefrontal cortex showed a more delayed and prolonged response, which is compatible with the attentional and modulatory role of this area (Lorenz et al., 2003). This finding is in agreement with our recent results on the temporal decomposition of the BOLD response following painful stimulation (Cauda et al., 2013a). We showed that the dorsolateral prefrontal cortex was characterized by a delayed and prolonged BOLD response, whereas the posterior insula was found to have a canonic HRF. These observations are also supported by the work of other groups (Mayhew et al., 2013).

The putamen is inconsistently observed in fMRI studies on pain (Tomycz and Friedlander, 2011) and is commonly described as involved in motor preparation and motor response to painful stimuli, although, recently, it has been proposed a role for the putamen in sensory aspects of pain perception (Starr et al., 2011). Our data show a delayed onset of the response in the putamen, which would support the view of this area as involved in motor response to potentially dangerous stimuli rather than in the sensory evaluation of sensory stimuli.

Overall our findings complement the idea that the detection of activity in some areas is more task dependent (e.g., the dorsolateral prefrontal cortex is typically found in studies involving attentional modulation of pain Porro et al., 1998; Peyron et al., 1999; Bantick et al., 2002) with the possibility that, in tasks not tapping on that particular function, responses can be still detected by using non- conventional approaches.

The IRS is however not free of pitfalls. This method has been originally used to analyze natural visual scenes (vision of a movie). Here, we provide a first attempt to apply it to punctate stimuli. Possible shortcomings of our approach rely in the confounding effect of habituation and in the possibility that the IRS picks up activity related to cognitive aspects of the elaboration of the stimulus, that is, not only the processing of the applied stimulus but also expectations about the incoming one. Our design did not allow us to control for this possibility. However, anticipation of the stimulus should occur in all participants at the same time in order to be included in the response to the stimulus. This possibility is unlikely because the ISI was jittering between 18 and 21 s in order to reduce chances of anticipation of the stimulus. Another possible confound may be represented by the synchronization between trials and co-occurring confounders, such as motion, respiration and heart rate. In order to account for this possibility, we regressed out such physiological confounds and then, after having re-analyzed the dataset, we compared the results of the two maps obtained with and without corrections for physiological noise. The results had a high level of agreement, therefore indicating that our findings were driven by the co-occurrence of noise with stimulus onset.

Might have the phase of the BOLD signal biased the results? We find this possibility unlikely. The interaction between resting state BOLD oscillations and activations related to the task is an issue for different kinds of fMRI approaches. In the GLM, spontaneous BOLD oscillations are not taken into account when the BOLD signal is modeled with a predictor, thus leaving open the possibility that results are also biased by the undergoing background activity. In our approach, each participant became the predictor for another one. Therefore there are two possibilities: if the spontaneous oscillation is different across subject, that is, it is random, it is highly likely that the response detected when using IRS is completely free from biases. Indeed, random noise would cancel out across different participants. However, also in the presence of the other possibility, namely that besides a random oscillation of the resting state networks there is a systematic oscillation (as proposed by Mayhew et al., 2013), such that the phase of some resting state networks systematically affects the response of the brain to external incoming stimuli, our results are still valid. In that case, if a significant interaction between resting state fluctuations and task related fluctuations modified our results, this modification would be due to the fact that a very similar resting state-task interaction took place in all participants. This kind of non- canonical response is exactly the focus of our study. Indeed, the complex relationship between spontaneous BOLD oscillations and task-related responses is now progressively being unveiled and the knowledge is still too sparse to make clear predictions. Indeed, it may be that the baseline cerebral blood flow and BOLD response have a strong effect on the BOLD response elicited by a concomitant stimulus (Davis et al., 1998; Hoge et al., 1999; Kastrup et al., 1999; Kim et al., 1999; Li et al., 1999; Corfield et al., 2001). As suggested by several other studies (Sapir et al., 2005; Ploner et al., 2006, 2010; Boly et al., 2007; Hesselmann et al., 2010; Pichè et al., 2010; Sadaghiani et al., 2010; Lee et al., 2011), it can be hypothesized that in our paradigm an interaction between large-scale brain networks (such as, for example, the DMN and fronto-parietal networks) and BOLD responses elicited by the stimuli may have led, in some specific areas, to a stimulus-locked response incorporating some of the temporal characteristics of the large-scale networks. That is, it is possible that a phenomenon of temporal synchronization between continuously ongoing brain fluctuations (related to large-scale brain networks) and BOLD responses to external stimuli exists. Such synchronization would give origin to an envelope constituted by the convolution between ongoing fluctuation and BOLD responses. If this is the case, in large-scale networks, the stimulus-related BOLD signal would be (at least partially) convolved with the network-specific resting time course and would generate a complex signal that incorporates some characteristics of both. However, this kind of complex interaction is likely to occur in any possible fMRI methodology and may represent a confounding factor that should be investigated more thoroughly in future studies, but that does not constitute a specific weakness of out method.

The IRS is not the only method available to analyze the data without imposing a priori knowledge about the shape of the BOLD response. Indeed, the need for methodologies that enable to study brain functions without imposing a priori knowledge about the BOLD response has led to an increasing number of innovative approaches (Tagliazucchi et al., 2010, 2012; Saka et al., 2012; Wu et al., 2013). Some recently proposed methods have used the detection of point processes to model the BOLD responses in both resting state and task-related activations. Another approach, the “total activation model,” was proposed by Karahanoglu et al. (2013). This method consists in finding an innovation system that should be sparse if the response evoked by the task (or at rest) is driven specifically by the task.

The results of the IRS analyses on mechanical punctate stimuli were compared to those obtained when using tactile stimuli in order to investigate if the IRS was able to differentiate between brain activities elicited by two different kinds of somatosensory stimuli. Our findings support this possibility by showing that the IRS detected both common, but, interestingly, also specific activations.

To summarize, our results show that the common picture that predictor-based fMRI studies give of pain processing can be expanded using innovative techniques which, vice versa, do not assume any a priori hypothesis of a BOLD envelope. Some of these areas show a gamma-like BOLD response but others are characterized by less-conventional hemodynamic envelopes.

However, two important caveats should be considered. First, the results of this study are not intended to provide a complete and exhaustive picture of all the brain areas that show a stimulus-locked response to pain but to point out the need of investigating pain-related activations using more advanced techniques (Brodersen et al., 2012) capable of detecting groups of areas characterized by non-conventional, prolonged or delayed BOLD responses. Second, we do not propose that the IRS is able to overcome shortcomings of the GLM and to constitute an alternative to it, but rather that the two techniques can lead to complementary results. Indeed, the IRS may be considered powerful enough to detect activity in areas with a canonical and a non-conventional response at the same time. However, in case of great variability in the BOLD response to the stimuli, the IRS may result less effective than the GLM.

The IRS method does not permit to disentangle different temporal BOLD response; thus, as in the GLM, all areas are visualized together. In this light, our findings can be complemented by those that we obtained using temporal clustering techniques (Cauda et al., 2013a). These techniques are able to decompose the stimulus-locked responses in a temporal window after stimulus presentation and cluster together voxels showing similar hemodynamic envelopes. However, whereas these techniques have the power to separate clusters showing unconventional responses, they are prone to the spatial and temporal variability of hemodynamic delay (Saad et al., 2001; Chang et al., 2008). Conversely IRS, which is based on a voxel-by-voxel comparison of homologous brain areas, shows results that are independent of the variability of hemodynamic delay. These two techniques can be used in synergy to clarify the different functional involvement of stimulus-locked brain areas in pain processing.

### Conflict of interest statement

The authors declare that the research was conducted in the absence of any commercial or financial relationships that could be construed as a potential conflict of interest.
